# High Resolution X-ray-Induced Acoustic Tomography

**DOI:** 10.1038/srep26118

**Published:** 2016-05-18

**Authors:** Liangzhong Xiang, Shanshan Tang, Moiz Ahmad, Lei Xing

**Affiliations:** 1 Department of Radiation Oncology, School of Medicine, Stanford University, Stanford, CA 94305, USA; 2 Center for Bioengineering and School of Electrical and Computer Engineering, University of Oklahoma, Norman, OK 73019, USA.

## Abstract

Absorption based CT imaging has been an invaluable tool in medical diagnosis, biology, and materials science. However, CT requires a large set of projection data and high radiation dose to achieve superior image quality. In this letter, we report a new imaging modality, X-ray Induced Acoustic Tomography (XACT), which takes advantages of high sensitivity to X-ray absorption and high ultrasonic resolution in a single modality. A single projection X-ray exposure is sufficient to generate acoustic signals in 3D space because the X-ray generated acoustic waves are of a spherical nature and propagate in all directions from their point of generation. We demonstrate the successful reconstruction of gold fiducial markers with a spatial resolution of about 350 μm. XACT reveals a new imaging mechanism and provides uncharted opportunities for structural determination with X-ray.

X-ray-induced acoustic waves has been observed^1^,^2^, while tomographic imaging with X-ray induced computed tomography (XACT) has been investigated in our previous experiments^3^. To induce acoustic waves, X-rays are absorbed by the excitation of inner-shell electrons and generate photoelectrons^4^,^5^. The excited electrons decay either by electromagnetic radiation, which may be reabsorbed, or by an Auger process. Auger electrons and photoelectrons transfer part of their kinetic energy to the surrounding medium by the production of cascades of secondary electrons. After multiple collisions, these electrons reach thermal equilibrium. The transfer of energy from the system of thermalized excited electrons to the tissue is governed by the electron-phonon interaction, which increases the temperature of the atomic system. The increase of the temperature in the irradiated volume is typically less than a millikelvin^6^, and the generated pressure waves are X-ray-induced acoustic (XA) signals (Fig. 1a). The X-ray-induced effect process is intrinsically three-dimensional (3D), as the XA waves are spherical in nature and propagate in all directions from their point of generation (Fig. 1b). The use of XA signals for volumetric imaging is uniquely advantageous: a single projection X-ray exposure is sufficient to generate acoustic signals in 3D space (Fig. 1c).

Mega electron-volts (MeV) energy X-rays with long pulse width (5 μs pulsed X-ray beams generated from a medical linear accelerator (LINAC)) was employed in our previous XACT imaging system. XA signal generation efficiency is relatively low and the spatial resolution is limited to millimeter level. While the XA signal produced by MeV X-rays opens the possibility of radiation dose monitoring during radiation therapy^3^, its application to diagnostic imaging is unlikely due to the poor image contrast and involvement of expensive LINAC equipment.

Our theoretical model predicts that the excitation pulse width is a crucial factor for the effective generation of ultrasonic waves. Here, we use ultrashort-pulsed X-rays (60 ns in pulse duration) to improve the XA signal generation efficiency as well as XACT imaging resolution. Our experimental results indicate that high resolution (350 μm) images can be readily achieved with the ultrashort-pulsed X-ray excitation.

## Results

### XACT imaging is only sensitive to the X-ray absorption

In XACT, we detect the acoustic signals generated by X-rays. When thermal confinement is satisfied, the physical models of XA signal generation and propagation for an arbitrary absorbing target with an arbitrary excitation source can be written as^7^,


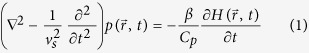


where *v*_*s*_ is the speed of sound, 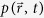 denotes the acoustic pressure rise at location 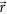 and time *t*, *β* is the thermal coefficient of volume, *C*_*p*_ denotes the specific heat capacity at a constant pressure, and 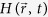 is the heating function. The left-hand side of equation (1) describes wave propagation in an inviscid medium, whereas the right-hand side represents the signal generating source. Equation (1) shows that the propagation of a XA pressure wave is driven by the first time derivative of the heating function 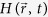. Therefore, time-invariant heating(i.e., the continuous X-ray source) does not generate an XA pressure wave, only time-variant heating(i.e., the pulsed X-rays source or the intensity-modulated X-ray sources) does.

For such a short X-ray pulse, the fractional volume expansion is negligible. Therefore the local pressure rise *p*_*0*_ can be said to occur immediately after the X-ray excitation and written as *p*_*0*_ = *Γη*_*th*_*μF.* Where *Γ* is Grueneisen parameter, *η*_*th*_is the percentage of absorbed energy that is converted to heat, *μ* denotes the X-ray absorption coefficient and *F* denotes the X-ray fluence (in joules per centimeter square). When focusing on the variation in the local absorption coefficient, we have Δ*p*_*0*_* /p*_*0*_ = Δ*μ/μ*, where Δ indicates a small variation in the modified variable. Considering that 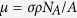, where *ρ* is the mass density, *σ* is the absorption cross section, and *N*_*A*_ and *A* are Avogadro number and atomic number, respectively, then the acoustic pressure variation Δ*p*_*0*_ is proportional to the variation of tissue characteristics Δ*σ*Δ*ρ*. Δ*ρ* denotes the change in tissue density, while Δ*σ* reflects the change in tissue compositions. Therefore, any fractional change in tissue characteristic translates into an equal amount of fractional change in the XA signal.

### XACT reduces radiation dose required for imaging a 3D subject

We calculated the minimal required dose for imaging a 100 μm-diameter breast calcification in a 16 cm-diameter breast phantom with XACT. The minimal dose is defined as the amount of dose needed to generate enough pressure amplitude over detector’s noise level while maintaining the spatial resolution at 100 μm. In our calculation, 5MHz ultrasound detector was applied and its noise was calculated by the noise equivalent pressure (NEP) model. In XACT, noise mainly arises from three sources: thermal acoustic noise from the medium, thermal noise from the ultrasonic transducer, and electronic noise from the amplifier. NEP can be expressed as follows^8^


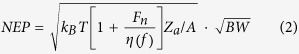


where *k*_*B*_ is the Boltzmann constant (1.38 × 10^−23^ J/K) and *T* is the absolute temperature of the medium in Kelvin. *F*_*n*_ denotes the noise factor of the amplifier and has a typical value of 2 over its bandwidth. For an ultrasound transducer with a center frequency of *f*_0_ and a detection bandwidth of Δ*f*, we assume that the detector efficiency is uniform such that 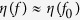, and 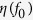 has a value of 0.5 (−3 dB). *Z*_*a*_ denotes the characteristic acoustic impedance of the medium (1.5 × 10^6^ Rayls for water), and *A* is the size of the detector. *BW* represents the bandwidth of an ultrasound detector. For a 5 MHz ultrasound transducer with a bandwidth of 100% that is approximately 3 cm^2^ in size at room temperature, the *NEP* is estimated as ∼22.7 × 10^−3^ Pa.

The minimal radiation dose for imaging a 100 μm breast calcification with XACT can be calculated as follows^8^,^9^,^10^


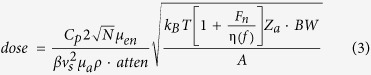


where *C*_*P*_ is the heat capacity (650 J/kg/K for calcium), *β* is 0.22 × 10^−4^ K^−1^ for calcium as the thermal coefficient. *ρ* (1.54 × 10^3^ kg/m^3^ for calcium) and *v*_*s*_ represent the density and sound speed of a calcification. *N* is the detector number on a hemisphere detector array, and *atten* is the acoustic attenuation from a calcification to an ultrasound detector. *μ*_*en*_ denotes the effective attenuation coefficient of breast tissue and *μ*_*a*_ is the mass attenuation coefficient of calcium. In our calculation, the minimal X-ray dose for imaging a 100 μm breast calcification with XACT is 0.36 mGy. The significant dose reduction may minimize the risk of radiation-induced cancer and benefit patients.

**Figure 2:**
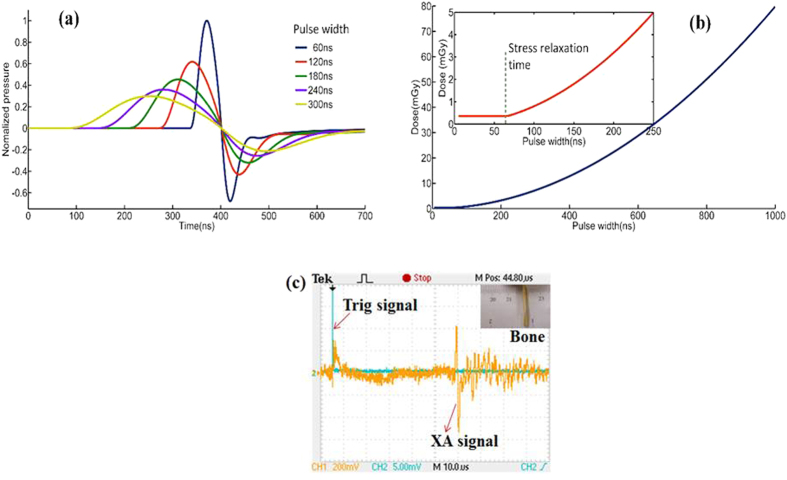
Computational and experimental XA signal generated by pulsed X-ray. (**a**) XA pressure generated from a pulsed X-rays for different pulse widths. (**b**) The dose sensitivity vs. pulse width. (**c**) The AX signals from a tiny piece of chicken bone were clearly detected.

**Figure 3:**
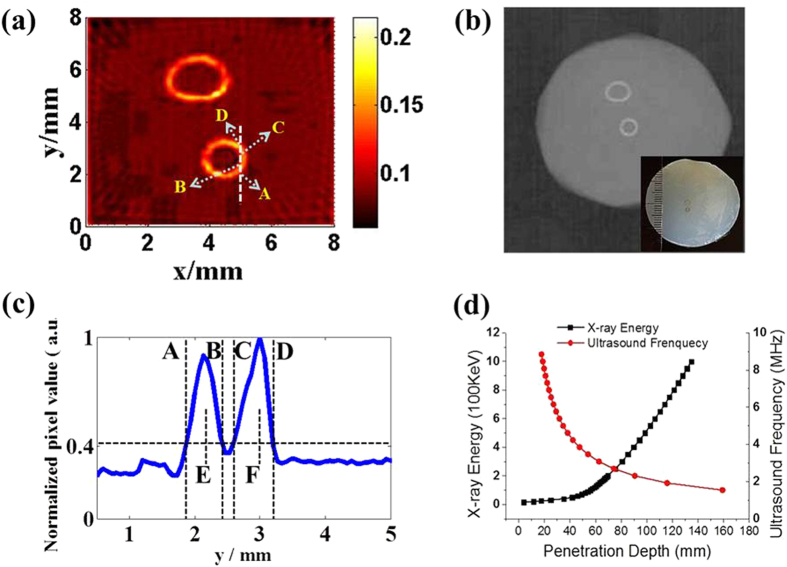
XACT preliminary experiment result. (**a**) Reconstructed XACT images of gold fiducial markers with different sizes by X-rays with a pulse width of 60 ns. (**b**) Conventional CT images. The inset figure at the bottom-right corner shows the sample picture. (**c**) Line profile of the reconstructed image shown in a with x = 4.9 mm. The spatial resolution of the XACT imaging system is determined about 0.35 mm. (**d**) The penetration depth of X-ray in different energy vs. the penetration depth of ultrasound in different frequency.

Simulations and experiments were conducted to show the performance of the XACT imaging: first, we compared the XA excitation efficiency at different X-ray pulse widths [Fig. 2a], then minimal X-ray dose was calculated for XACT imaging [Fig. 2b], finally the XA signal was recorded from a piece of chicken bone with a length and width of 20 mm × 2.85 mm. A major XA peak with 1.9 μs duration is readily observed in the signal [Fig. 2c], which is equivalent to the acoustic time delay between the front and rear boundary of the chicken bone, which corresponds to a size of 2.85 mm. Finally, high-resolution XACT images of gold fiducial markers with sub-mm sizes in phantom were reconstructed [Fig. 3a].

Figure 3 shows the two dimensional XACT image, CT image, and the photo of the gold fiducial markers in phantom. In the reconstructed XACT image shown in Fig. 3a, the two rings were clearly detected. We found that the recovered size and shape of the gold fiducial rings was in good agreement with the CT image and the phantom photograph. The cross section of one gold fiducial ring was imaged to estimate the spatial resolution of the XACT imaging. Figure 3c plots the normalized intensity profile of the cross-section image at x = 4.9 mm in Fig. 3a. The 40.5% amplitude line intercepts the profile at points A, B, C, and D. The respective centers of the two absorption peaks are located at points E and F. The spatial resolution is estimated according to the Rayleigh criterion^11^,^12^. The two sources can no longer be clearly distinguished when point B touches point C in Fig. 3c. Therefore, the minimum distinguishable distance, R, between the two sources is approximately R = |EB| + |CF| − 2r, where 2r is the thickness of the target measured to be 150 μm. The spatial resolution is thus determined to be 350 μm.

## Discussion

We have developed a high resolution XACT by the ultrashort-pulsed X-ray excitation. We presented the results of our theoretical and experimental investigations on this new imaging modality. The XA signals from a small piece of chicken bone in phantom were clearly detected and high resolution XACT imaging for gold fiducial markers has been demonstrated in the experiments. The current pulsed X-ray source has a high energy (270kVp) and is thus limited to imaging high density material. Soft tissue imaging with low energy X-rays will be investigated in the future experiments.

Based on our theoretical model and calculations, the pulse width of excitation X-rays is a crucial factor for the effective generation of XA waves^13^. Our theoretical analysis shows that the resultant XA pressure is proportional to the time derivative of the excitation pulse^8^. Compared to our previous XACT system, the implementation of the 60 ns pulsed X-rays resulted in a conversion efficiency increase of more than 3 orders of magnitude. The capability of 3D acoustic image generation using a single X-ray projection is unique and may revolutionize some clinical imaging procedures, such as breast imaging, in the future.

It is important to emphasize that the XACT signal depends only on the X-ray absorption. Therefore, a change in tissue characteristic translates into a proportional change in the acoustic signal. This unique feature may enable XACT as an *in vivo* bone densitometry tool to quantitatively measure the bone mineral density at the earliest stage.

The acoustic flight time in pulsed excitation provides depth information about the absorbing targets. In general, the axial resolution is jointly determined by the X-ray pulse width, and detection bandwidth of the ultrasonic transducer. In our experiments, a 2.25 MHz ultrasound transducer was used, which determined the imaging resolution with *R* = *0.88 v*_*s*_* /f*_*max*_ of about 0.30 mm^14^. If a higher frequency transducer and a narrower pulsed X-ray source are employed in experiments, sub-micrometer spatial resolution should be achievable, which may provide a new approach of microstructural determination with X-rays.

As compared to the non-ionizing radiation imaging techniques^14^,^15^,^16^,^17^,^18^,^19^,^20^, such as optical imaging modalities or laser-based photoacoustic imaging, a downside of XACT is that it delivers a low dose radiation to the patient to gain information at depth in imaging. In reality, whether X-ray imaging should be used is a clinical decision with consideration of potential benefits and risks. As depicted in Fig. 3d 21, a useful benefit of XACT is its ability of visualizing deep-seated micro-calcifications that are beyond the limitation of emerging non-ionizing imaging strategies. Thus XACT has potential be translated into clinical practice in the future.

## Methods

**Figure 4:**
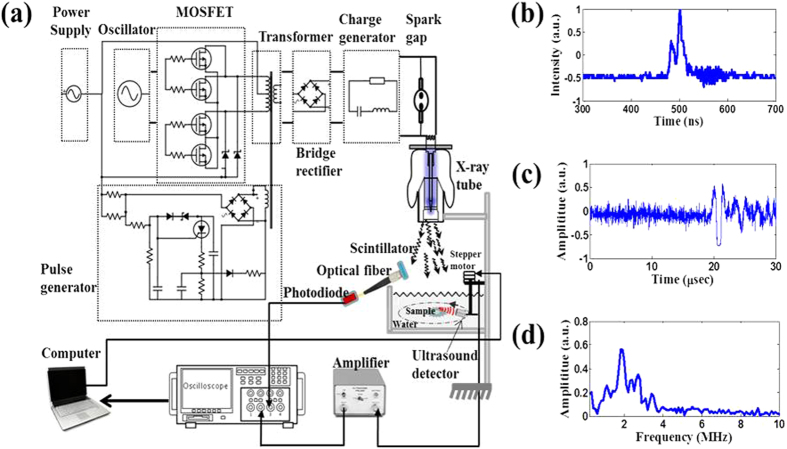
XACT experiment setting. (**a**) Schematic diagram of the experimental setup. (**b**) 60 ns pulsed X-rays detected by the photodiode/scintillator combo for triggering the acoustic data acquisition. (**c**) A typical X-ray-induced acoustic signal recorded in time domain. (**d**) *FFT* spectrum of the detected XA signal.

Figure 4a shows a schematic of an XACT system. It comprises three major hardware components: (i) an X-ray tube to produce ultrashort-pulsed X-rays for the XA signal generation, (ii) a low noise ultrasound detector, and (iii) a signal processing and data acquisition system. The X-ray generator (XRS-3, Golden Engineering Inc., IN, USA) can provide ultrashort pulsed X-rays with 60 ns pulse width operated at an energy of 270 kVp. The pulse width is measured with a photodiode/scintillator combo shown in Fig 4b. Also, the pulse repetition frequency is 15 Hz. The radiation dose of each X-ray pulse is about 0.03 mGy. The detected X-ray pulse was used as a trigger signal to synchronize the ultrasound detector and oscilloscope for recording the X-ray-induced acoustic signal. The acoustic signal was captured by the ultrasound transducer (V397-SU, Olympus-NDT, Waltham, MA, USA) which has a central frequency of 2.25 MHz and a diameter of 29 mm. The transducer driven by a computer-controlled step motor, to scan around the sample, detected the XA signals in the imaging plane at each scanning position. A pulse preamplifier (PREAMP2-A, Ultratek Inc, Concord, CA, USA) with bandwidth of 20 KHz–30 MHz at −3 dB and gain range of 10–60 dB was used to amplify the acoustic signal. Signals were recorded and averaged 16 times by the oscilloscope (Tektronix TDS 1002C-EDU, Richardson, Texas, USA). A sampling rate of 100 MHz was used to record 2500 data points at each view angle. All control codes were written using labview graphic programming language (National Instruments, TX, USA). One set of data incorporated 200 positions as the receiver moved 360°. The XACT image was then reconstructed with the filtered back projection algorithm^3^,^22^,^23^. Phantoms made of 5% gelatin and 95% water were used throughout the experiment. The sample is submerged in the water for coupling the acoustic signal from the target to the detector. Figure 4c shows the temporal profile of a typical XA signal detected by the system. The fast Fourier transform (FFT) was performed and the ultrasound frequency spectrum data *h ( f)* is shown in Fig. 4d.

## Additional Information

**How to cite this article**: Xiang, L. *et al.* High Resolution X-ray-Induced Acoustic Tomography. *Sci. Rep.* **6**, 26118; doi: 10.1038/srep26118 (2016).

## Figures and Tables

**Figure 1:**
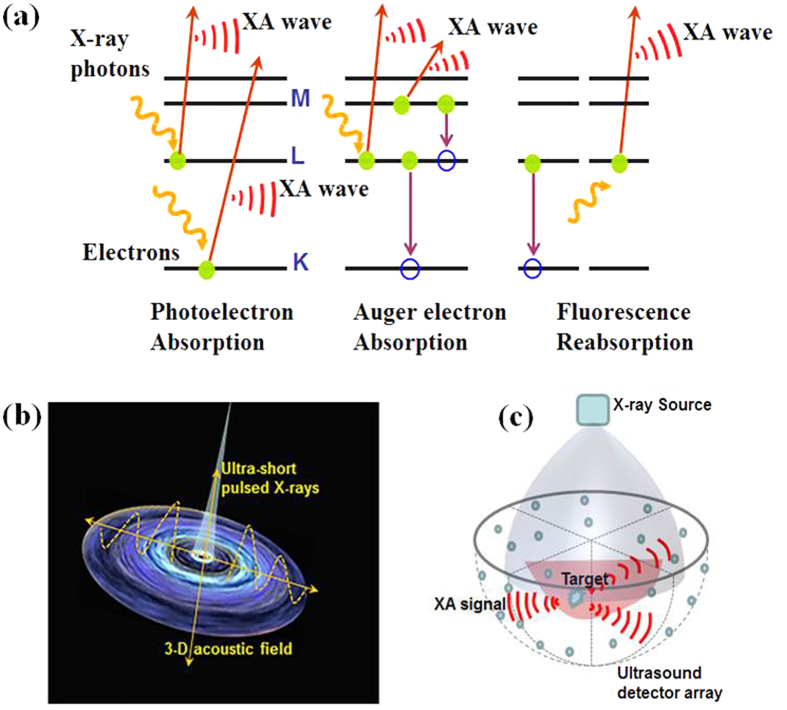
Principles of XA signal generation and detection. (**a**) Schematic of the main processes which contribute to XA signals, namely photoelectrons, Auger electrons, and reabsorbed radiation. (**b**) Schematic of the 3D acoustic field generated by ultrashort-pulsed X-rays. (**c**) Schematic diagrams of 3D XACT.
